# Protective effects of alpha phenyl-tert-butyl nitrone and ascorbic acid in human adipose derived mesenchymal stem cells from differently aged donors

**DOI:** 10.18632/aging.101035

**Published:** 2016-09-15

**Authors:** Adiv A. Johnson, Yahaira Naaldijk, Christian Hohaus, Hans Jörg Meisel, llona Krystel, Alexandra Stolzing

**Affiliations:** ^1^ Department of Ophthalmology, Mayo Clinic, Rochester, MN 55905, USA; ^2^ Translational Centre for Regenerative Medicine (TRM), Leipzig University, Leipzig, Germany; ^3^ Department of Neurosurgery, BG Clinic Bergmannstrost Halle, Germany; ^4^ Loughborough University, Centre for Biological Engineering, Wolfson School, Loughborough, UK; ^5^ Fraunhofer Institute for Cell Therapy and Immunology, Leipzig, Germany

**Keywords:** ADSCs, antioxidants, aging, comet assay, DNA damage, DNA repair

## Abstract

Adipose-derived mesenchymal stem cells (ADSCs) are multipotent stem cells that promote therapeutic effects and are frequently used in autologous applications. Little is known about how ADSCs respond to genotoxic stress and whether or not donor age affects DNA damage and repair. In this study, we used the comet assay to assess DNA damage and repair in human ADSCs derived from young (20-40 years), middle-aged (41-60 years), and older (61+ years) donors following treatment with H_2_O_2_ or UV light. Tail lengths in H_2_O_2_-treated ADSCs were substantially higher than the tail lengths in UV-treated ADSCs. After 30 minutes of treatment with H_2_O_2_, ADSCs preconditioned with alpha phenyl-tert-butyl nitrone (PBN) or ascorbic acid (AA) showed a significant reduction in % tail DNA. The majority of ADSCs treated with PBN or AA displayed low olive tail movements at various timepoints. In general and indicative of DNA repair, % tail length and % tail DNA peaked at 30 minutes and then decreased to near-control levels at the 2 hour and 4 hour timepoints. Differently aged ADSCs displayed comparable levels of DNA damage in the majority of these experiments, suggesting that the age of the donor does not affect the DNA damage response in cultured ADSCs.

## INTRODUCTION

Adipose-derived mesenchymal stem cells (ADSCs) are multipotent cells that can differentiate into adipocytes, chondrocytes, and osteoblasts [1]. They have also been reported to differentiate into other cell types, including skeletal and cardiac muscle cells [1, 2]. ADSCs are an ample source of adult stem cells that, unlike mesenchymal stem cells (MSCs) derived from bone marrow, can be procured by a simple and minimally invasive procedure [3]. ADSCs can be stably expanded under basic culture conditions and exhibit a low risk of contamination [3]. Patients receiving a transplant of their own ADSCs are unlikely to experience immune rejection following transplantation [4] and ADSCs secrete factors that promote healing and regeneration [1, 5, 6]. The conditioned medium derived from ADSCs is also therapeutic and has been shown to exert protective effects in chondrocytes, such as the reduction of oxidative and inflammatory stress [7]. As such, ADSCs represent an attractive therapeutic source of adult stem cells and have been the focus of many preclinical and clinical studies geared towards a variety of applications [8, 9]. ADSC-centered therapies have been shown to be promising for a variety of clinical applications, including neurodegenerative disorders [10], arthritis [11], wound healing [12], diabetes [13, 14], autoimmune disease [4, 14], plastic surgery [15], and many others.

Given the therapeutic potential of ADSCs, strategies that improve their efficacy are of clinical utility. It was previously shown that in vitro culturing of ADSCs with a growth medium low in calcium and supplemented with the antioxidants N-acetyl L-cysteine and L-ascorbic acid-2-phosphate prolonged their lifespan and accelerated their growth [16]. A separate study reported that exposure of ADSCs to the same antioxidants (N-acetyl-L-cysteine and L-ascorbic acid-2-phosphate) promotes entry into the S phase of the cell cycle and results in more rapid cell proliferation [17]. These antioxidants were also reported to mediate positive changes in cytokine expression, telomere length, osteogenesis, adipogenesis, and chondrogenesis [17]. In addition to being influenced by antioxidants [16, 17], ADSCs can induce antioxidant effects under a variety of conditions [12, 18].

Although ADSCs seem to significantly affect and be affected by antioxidants, very little is known about their ability to respond to various stressors, such as genotoxic stress. Genotoxic stress can be caused by ionizing radiation, ultraviolet light, reactive oxygen species, and chemical mutagens [19]. This stress can cause DNA damage and lead to genomic instability, which is one of the major hallmarks of aging [20]. Treatment of ADSCs with the chemical carcinogenic 4-nitroquinoline-1-oxide was found to trigger their terminal differentiation into adipocytes [21]. A separate study reported that exposing ADSCs to GC-enriched cell free DNA induced both single-and double-strand breaks in DNA and caused an increase in the expression of repair and antiapoptotic genes [22]. Exposure to this GC-rich extracellular DNA was also reported to stimulate the differentiation of ADSCs into adipocytes [22].

The production of stem cells for therapies requires rigorous quality control testing, including testing of genetic stability [23]. DNA damage can cause cell apoptosis and senescence [24], which in turn can alter the secretome [25] and potentially hazard surrounding tissue. Consequently, knowledge regarding how ADSCs respond to DNA damage and strategies to minimize genotoxic stress is therapeutically relevant. To this effect, we investigated DNA damage and DNA repair in response to hydrogen peroxide (H_2_O_2_) and ultra-violet (UV) light in ADSCs derived from differently aged donors. We also assessed the ability of ascorbic acid (AA) and alpha phenyl-tert-butyl nitrone (PBN) to protect against DNA damage in ADSCs derived from differently aged sources. AA, or Vitamin C, is a well characterized antioxidant known to affect DNA repair [26] and minimize lipid, DNA, and protein oxidation [27]. PBN is a potent free radical scavenger and spin trap agent reported to mediate numerous beneficial health effects, such as the prevention of light-induced retinal degeneration [28], the reduction of mechanical allodynia [29], and the inhibition of painful peripheral neuropathy [30]. PBN has also been shown to reverse mitochondrial decay in a mouse model Chagas’ disease, suggesting that PBN works in part by targeting mitochondria [31].

In this study, we report basal levels of DNA damage and DNA repair in ADSCs following treatment with UV or H_2_O_2_. We find that that both AA and PBN help attenuate H_2_O_2_-induced DNA damage in ADSCs derived from differently aged sources.

## RESULTS

### ADSCs from differently aged donors are susceptible to DNA damage in response to UV light and H_2_O_2_

**Figure 1:**
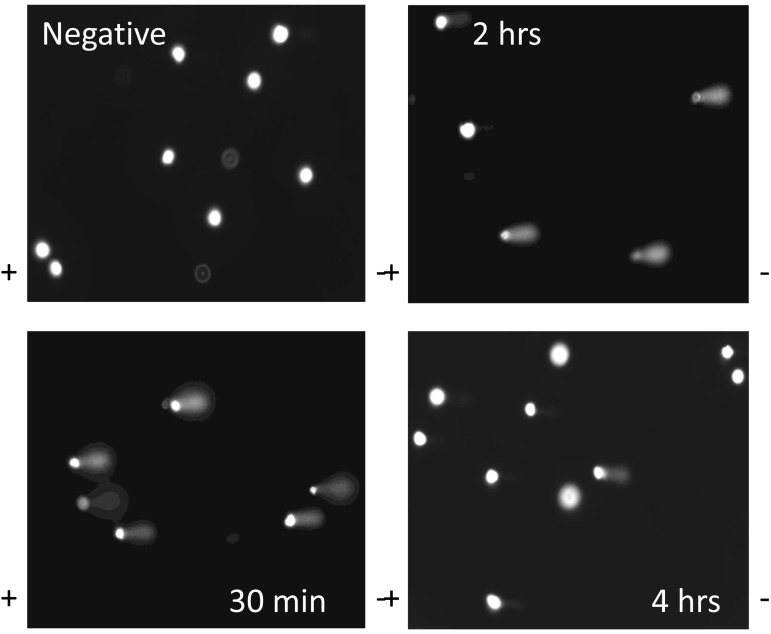
DNA comets after treatment of ADSCs with H_2_O_2_ Representative slides of DNA comets from human ADSCs treated with H_2_O_2_ for 30 minutes are shown. Slides are shown for ADSCs untreated with H_2_O_2_ (negative control) or H_2_O_2_-treated ADSCs at the 30 minute, 2 hour, and 4 hour timepoints. DNA comet tails were notably pronounced at the 30 minute and 2 hour timepoints. The comet tails decreased in length at the 4 hour timepoint.

To gain insight into the sensitivity of ADSCs to genotoxic stress, we subjected human ADSCs from young (20-40 yrs), middle-aged (41-60 yrs), or older (61+ yrs) donors to H_2_O_2_ or UV light. As described in the Methods, comet assays were performed and DNA damage levels quantified by measuring tail length at different timepoints (Fig. 1). Tail length is defined as the distance of DNA migration from the nuclear body of the comet and is an indicator of the extent of DNA damage. Comets with clearly defined tails after treatment with H_2_O_2_ at 30 min, 2 hr, and 4 hr timepoints are shown in Figure 1. The controls, which were not treated with H_2_O_2_, showed no comet tails (Fig. 1).

**Figure 2:**
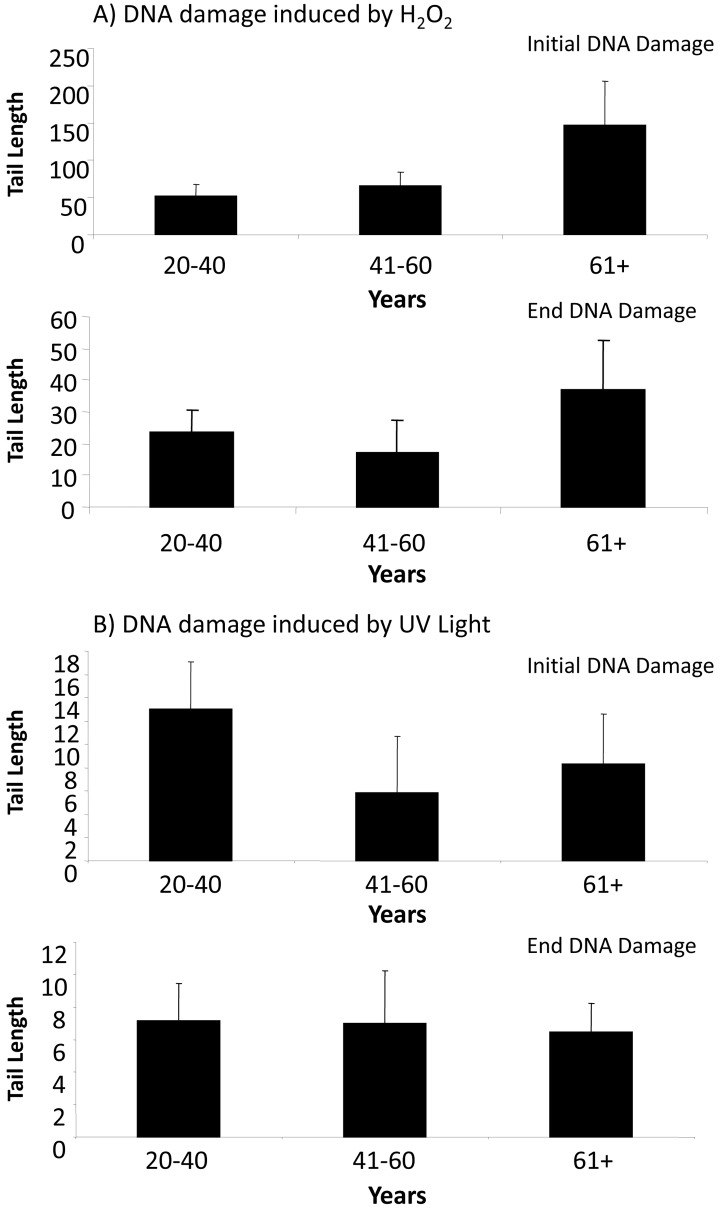
DNA damage in ADSCs treated with H_2_O_2_ or UV light (**A**) Tail length was quantified in ADSCs derived from young (20-40 years), middle-aged (41-60 years), and older (61+ years) after treatment with H_2_O_2_ for 30 minutes. Tail length was measured at the 30 minute (initial DNA damage) and 4 hour (end DNA damage) timepoints. Although tail length was longer in the ADSCs derived from 61+ year old donors, the tail length in differently aged ADSCs was statistically comparable. (**B**) Tail length was measured in ADSCs from differently aged sources following treatment with UV light for 30 minutes. Tail length was statistically comparable in ADSCs derived from young, middle-aged, and older donors. In comparison to ADSCs treated with H_2_O_2_, tail length was drastically shorter in ADSCs treated with UV light.

In response to treatment with H_2_O_2_, ADSCs derived from older donors exhibited an increase in tail lengths compared to ADSCs derived from middle-aged or younger donors (Fig. 2A). Although this was true for both initial DNA damage (measured at 30 min) and end DNA damage (measured at 4 hr) (Fig. 2A), this increase was not statistically significant. Tail lengths in ADSCs derived from young, middle-aged, and older donors were statistically comparable (Fig. 2A). Tail length was notably reduced at the 4 hr timepoint compared to the 30 min timepoint (Fig. 2A), indicative of DNA repair mechanisms at work.

In response to UV light, ADSCs from younger donors displayed the longest tail length at the initial DNA damage timepoint. This difference was not statistically significant, however, and the measured tail lengths at both the initial DNA damage and end DNA damage timepoints were comparable for ADSCs from differently aged donors. At both the initial DNA damage and end DNA damage timepoints, the tail length in ADSCs treated with UV light was drastically smaller than the tail length in ADSCs treated with H_2_O_2_ (Fig. 2).

### Both PBN and AA reduce H_2_O_2_-induced DNA damage in ADSCs derived from differently aged donors

**Figure 3:**
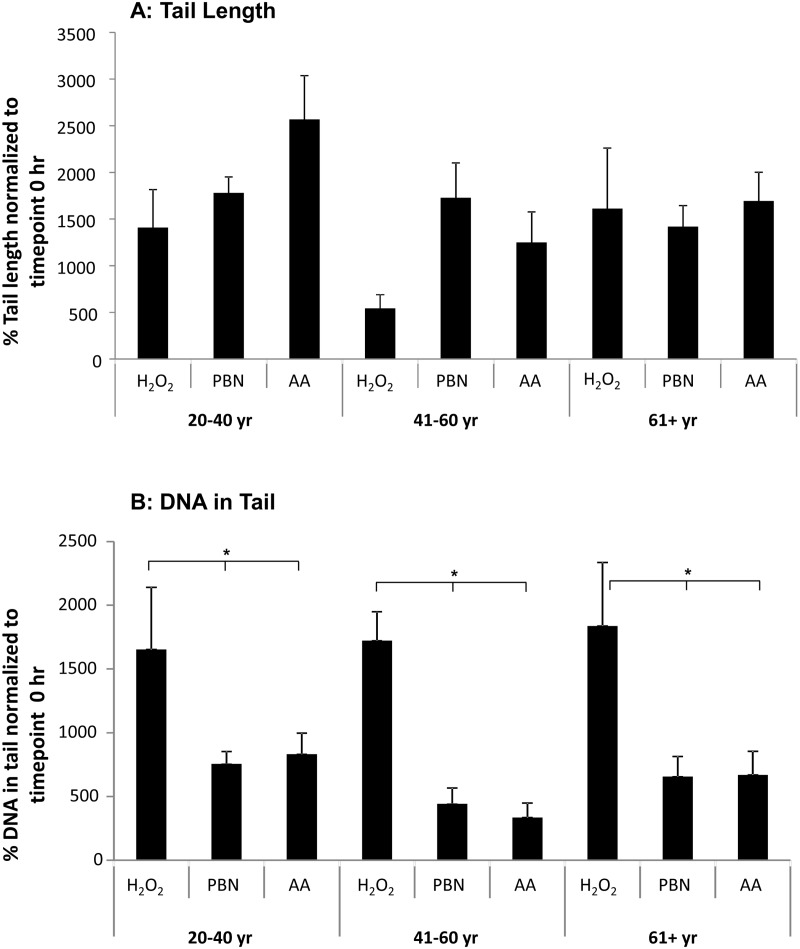
PBN and AA reduce DNA damage in H_2_O_2_-treated ADSCs ADSCs from young, middle-aged, and older sources were preincubated for 24 hours with either PBN or AA and then treated with H_2_O_2_ for 30 minutes. (**A**) ADSCs preincubated with either PBN or AA displayed comparable % tail lengths to the controls, which were treated with H_2_O_2_ alone and not preincubated with PBN or AA. (**B**) ADSCs preincubated with either PBN or AA showed a significant reduction in % tail DNA compared to the controls. This was true for ADSCs derived from each age group. * indicates p < 0.05.

Since the tail lengths observed in response to UV light were extremely short compared to the DNA damage observed in response to H_2_O_2_ (Fig. 2), we were curious if treatment with specific agents could attenuate H_2_O_2_ -induced DNA damage in ADSCs. To assess this, we tested the ability of both PBN and AA to reduce levels of DNA damage in ADSCs subjected to 30 min of treatment with H_2_O_2_ (Fig. 3).

We find that treatment with either PBN or AA failed to significantly affect % tail length in H_2_O_2_-treated ADSCs (Fig. 3A). This prompted us to next measure % tail DNA, a comet assay parameter that is linearly related to the frequency of DNA breaks. Unlike tail length (Fig. 2A), both PBN and AA significantly reduced the % tail DNA in ADSCs from young, middle-aged, and older sources after 30 min of treatment with H_2_O_2_ (Fig. 3B).

**Figure 4:**
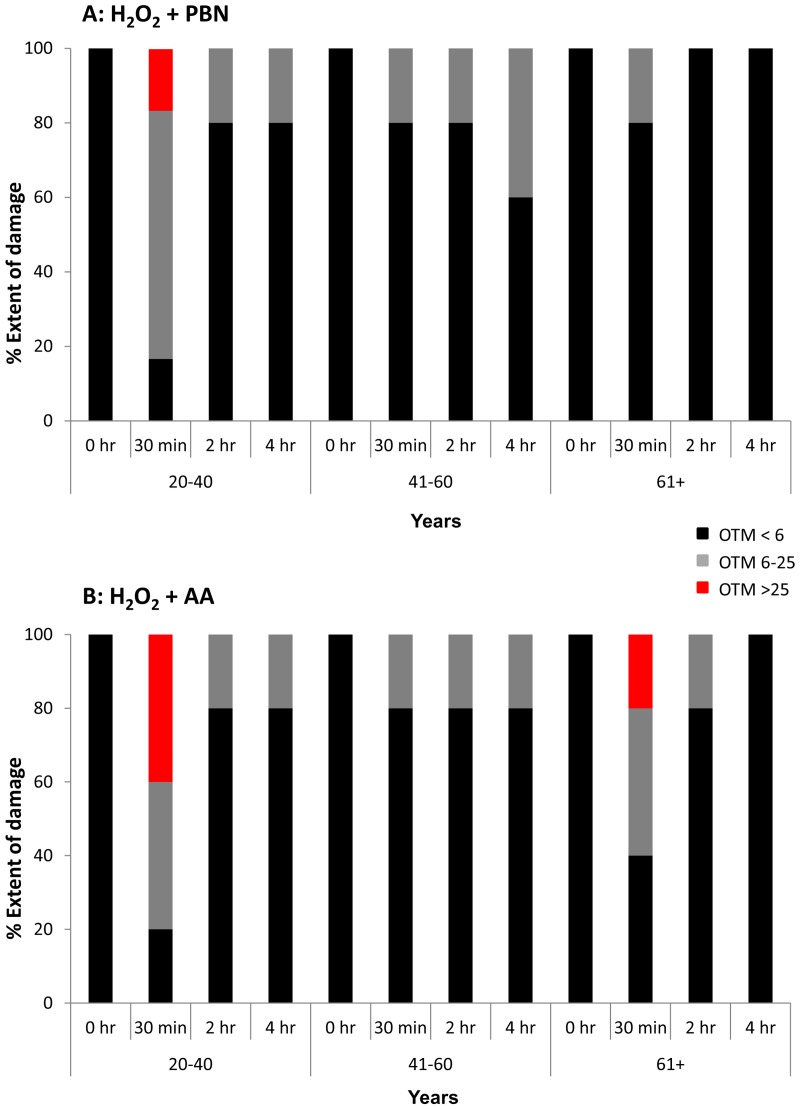
OTM was low in the majority of H_2_O_2_-treated ADSCs preincubated with PBN or AA Olive tail movement (OTM) at the 0 hour, 30 minute, 2 hour, and 4 hour timepoints was measured in H_2_O_2_-treated ADSCs additionally treated with PBN or AA. The % of the ADSC population showing an OTM < 6 (black), an OTM between 6 and 25 (grey), or an OTM > 25 (red) is shown at each timepoint. (**A**) In general, the OTM was quite low (< 6) in ADSCs treated with PBN. Higher OTMs were found at the 30 minute timepoint in ADSCs derived from 20-40 year old donors. (**B**) Similarly, the majority of ADSCs treated with AA showed an OTM < 6. Higher OTMs were observed at the 30 minute timepoint in ADSCs derived from 20-40 year old and 61+ year old donors.

We next measured the olive tail moment (OTM) in ADSCs treated with H_2_O_2_ and PBN (Fig. 4A) or H_2_O_2_ and AA (Fig. 4B). The OTM is a comet assay parameter equal to the product of tail length and the fraction of total DNA within the tail. At the 0 hr timepoint,100% of the measured OTM was very low (<6), which is indicative of little to no DNA damage (Fig. 4). In general, the OTM in ADSCs derived from differently aged populations at each timepoint was quite low (<6). Some portions of the population showed mid-range OTMs between 6 and 25. At the 30 min timepoint, a portion of ADSCs from young sources displayed OTMs >25 for both the H_2_O_2_ and PBN (Fig. 4A) and H_2_O_2_ and AA (Fig. 4B) groups. At the 30 min timepoint, a portion of ADSCs derived from humans aged 61+ also displayed OTMs >25 for the H_2_O_2_ and AA group (Fig. 4B). In general, however, ADSCs treated with H_2_O_2_ and either PBN or AA showed minimal OTMs (Fig. 4), which is indicative of minimal DNA damage in the presence of these protective agents.

### PBN and AA promote DNA damage repair in ADSCs from differently aged sources

In Figure 2, we found that % tail length dramatically decreased at the 4 hr timepoint compared to the 30 min timepoint for H_2_O_2_-treated ADSCs. This is indicative of endogenous DNA repair mechanisms alleviating the DNA damage induced by H_2_O_2_. We sought to further characterize these DNA repair mechanisms by assessing DNA damage parameters over time in ADSCs treated with PBN or AA.

**Figure 5:**
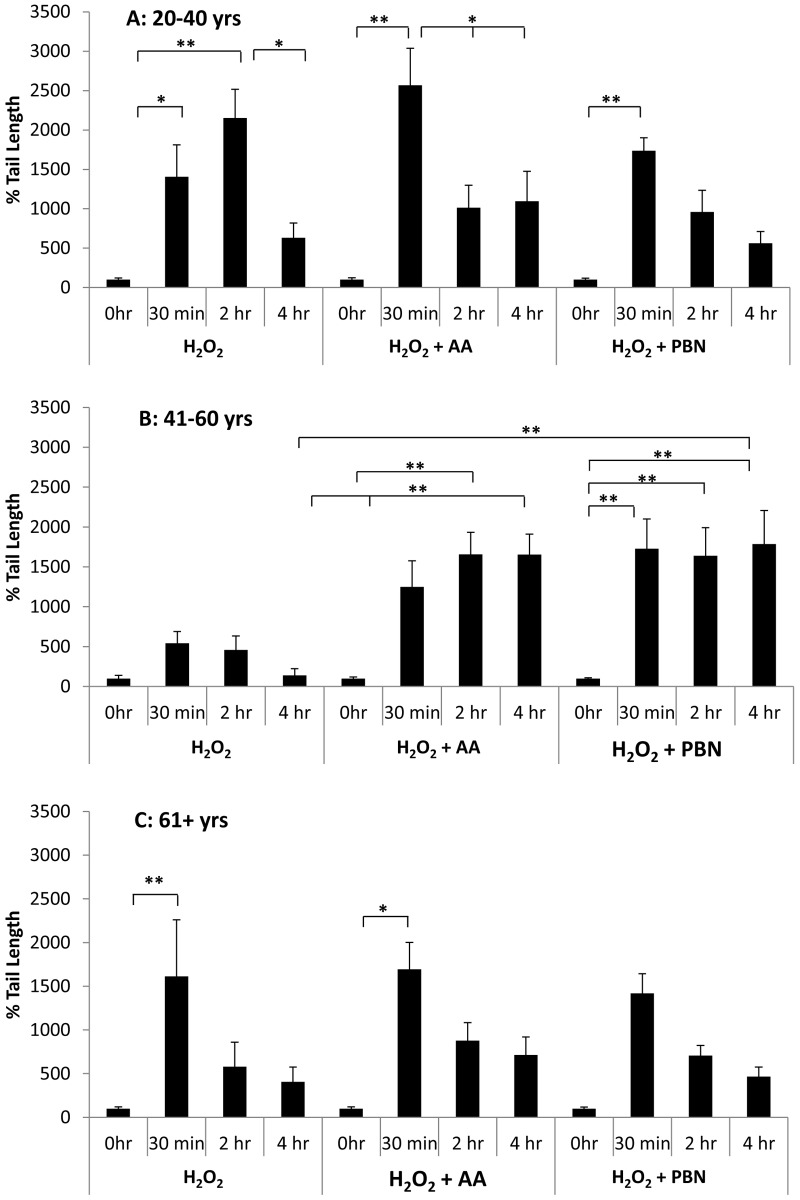
Changes in % tail length over time in ADSCs treated with H_2_O_2_ % tail length was measured in ADSCs derived from 20-40 year old donors (**A**), 41-60 year old donors (**B**), or 61+ year old donors (**C**) at the 0 hour, 30 minute, 2 hour, and 4 hour timepoints after treatment with H_2_O_2_ for 30 minutes. % tail length was measured in ADCSs treated with H_2_O_2_ alone or in ADSCs additionally treated with AA or PBN. * indicates p < 0.05 and ** indicates p < 0.005.

We find that, in young ADSCs (Fig. 5A), H2O2 induces a significant increase in % tail length at the 30 min and 2 hr timepoints. After 4 hrs, the % tail length significantly drops to a level similar to the 0 hr timepoint control (Fig. 5A). In response to treatment with AA, % tail length again significantly increases at the 30 min timepoint. % tail length then significantly decreases at the 2 hr and 4 hr timepoints, returning to levels statistically comparable to the control (Fig. 5A).

After treatment with PBN, % tail length significantly increases at the 30 min timepoint but then decreases at the 2 hr and 4 hr timepoints (Fig. 5A). For middle-aged ADSCs (Fig. 5B), treatment with H_2_O_2_ alone did not significantly increase % tail length at any of the timepoints measured. In response to treatment with AA or PBN in addition to H_2_O_2_, however, a significant increase in % tail length was observed at all timepoints compared to 0 hr controls (Fig. 5B). Moreover, the % tail length in ADSCs treated with H_2_O_2_ and AA or H_2_O_2_ and PBN was greater than the % tail length in ADSCs treated with H_2_O_2_ alone at the 4 hr timepoint (Fig. 5B). In older ADSCs, the % tail length significantly increased at the 30 min timepoint in response to H_2_O_2_ or H_2_O_2_ and AA (Fig. 5C). Otherwise, the % tail lengths measured were statistically comparable to the 0 hr timepoint (Fig. 5C).

**Figure 6:**
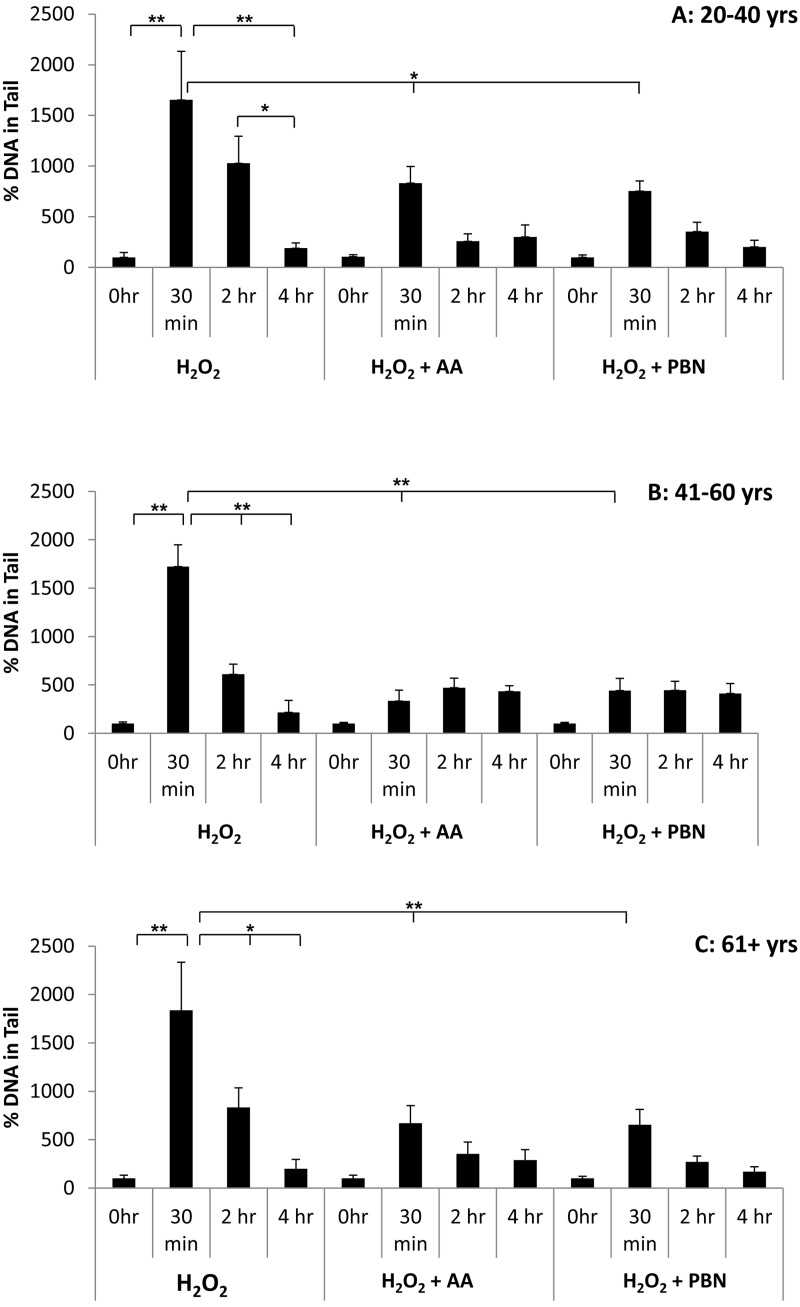
Changes in % tail DNA over time in ADSCs treated with H_2_O_2_ % tail DNA was measured in ADSCs derived from 20-40 year old donors (**A**), 41-60 year old donors (**B**), or 61+ year old donors (**C**) at the 0 hour, 30 minute, 2 hour, and 4 hour timepoints after treatment with H_2_O_2_ for 30 minutes. % tail DNA was measured in ADCSs treated with H_2_O_2_ alone or in ADSCs additionally treated with AA or PBN. * indicates p < 0.05 and ** indicates p < 0.005.

We next quantified % tail DNA in differently aged ADSCs treated with H_2_O_2_, H_2_O_2_ and AA, or H_2_O_2_ and PBN at the 0 hr, 30 min, 2 hr, and 4 hr timepoints (Fig. 6). In response to H_2_O_2_, a significant increase in % tail DNA was observed at the 30 min and 2 hr timepoints for ADSCs derived from younger donors. % tail DNA had returned to near-basal levels after 4 hr (Fig. 6A). Compared to ADSCs that received H_2_O_2_ alone, the % tail DNA was significantly lower in the ADSCs that received AA or PBN at the 30 min timepoint (Fig. 6A). For ADSCs obtained from middle-aged (Fig. 6B) or older (Fig. 6C) donors, % tail DNA was comparable at all timepoints for the ADSCs treated with either AA or PBN (Fig. 6B, C). In contrast, the % tail DNA at the 30 min timepoint was significantly increased in ADSCs treated with H_2_O_2_ alone (Fig. 6B, C). Compared to the group treated with H_2_O_2_, the % tail DNA was significantly lower in the groups additionally treated with AA or PBN at the 30 min timepoint (Fig. 6B, C).

## DISCUSSION

In this study we employed the comet assay to assess DNA damage in ADSCs treated with either H_2_O_2_ or UV light. We measured DNA damage and assessed DNA repair in ADSCs derived from differently aged sources as well as in ADSCs treated with either PBN or AA.

To our surprise, the tail lengths in ADSCs treated with H_2_O_2_ were substantially higher than the tail lengths in ADSCs treated with UV light. This was the case for ADSCs from differently aged sources at both the 30 minute and 4 hour timepoints of measurement (Fig. 2). The energy provided from UV light directly damages DNA by introducing cytotoxic DNA lesions, such as pyrimidine 6-4 pyrimidone photoproducts, their Dewar isomers, and cyclobutane-pyrimidine dimers. UV light can induce DNA strand breaks and has also been reported to stimulate the generation of free radicals [32, 33]. H_2_O_2_ is a strong oxidizer and a reactive oxygen species that causes free-radical induced DNA lesions and breaks [19, 34]. The molecular mechanism of H_2_O_2_-induced senescence in ADSCs has been partially elucidated and involves the p53/p21/pRb and p38MAPK/MAPKAPK-2 pathways, both of which lead to DNA damage response activation [35]. Since tail length is a parameter used to generally evaluate the extent of DNA damage, our data (Fig. 2) would suggest that human ADSCs are more sensitive to reactive oxygen species-induced DNA damage than DNA damage caused by direct interaction with UV light. In response to either H_2_O_2_ or UV light, the tail length was notably decreased at the 4 hour timepoint compared to the 30 minute timepoint. Since the ADSCs were subjected to a genotoxic stressor for 30 minutes, this demonstrates that endogenous DNA repair mechanisms are at work and that, by hour 4, a significant portion of the DNA damage was rectified.

Since tail length was greater in H_2_O_2_-treated ADSCs than in UV-treated ADSCs, we proceeded to investigate the ability of PBN and AA to attenuate DNA damage in H_2_O_2_-treated cells. Although treatment of ADSCs with PBN or AA failed to significantly affect % tail length in H_2_O_2_-treated ADSCs, treatment with PBN or AA did significantly attenuate % tail DNA in ADSCs from differently aged sources (Figs. 3 and 6). % tail DNA directly correlates with DNA break frequency and, as such, our data would indicate that both PBN and AA are capable of reducing the frequency of DNA breaks in H_2_O_2_-treated ADSCs. Genotoxic stress has been previously reported to trigger the differentiation of ADSCs into adipocytes [21, 22] and complications in DNA damage and repair have been reported following allogeneic transplantation with stem cells [36]. Therefore, the basal levels of DNA damage in MSCs may affect their efficiency and therapeutic potential following transplantation. Our data argue that preincubation with either PBN or AA is an effective method to reduce DNA damage in cultured MSCs and may be an efficacious means of optimizing the genomic integrity of transplanted MSCs. Advocating this, the OTM was very low in the majority of H_2_O_2_-treated ADSCs preincubated with either PBN or AA (Fig. 4). Some cells, however, did display a higher OTM. This indicates that the beneficial effects of PBN and AA do not necessarily extend to all cells within a treated population.

In response to H_2_O_2_, % tail DNA peaked at the 30 minute timepoint and then gradually decreased at the 2 hour and 4 hour timepoints. By hour 4, % tail DNA was comparable to controls. This was true of ADSCs obtained from young, middle-aged, and older donors (Fig. 6). In H_2_O_2_-treated ADSCs, % tail length tended to increase at the 30 minute timepoint and then drop down by the 4 hour timepoint (Fig. 5). These data demonstrate that ADSCs possess robust DNA repair mechanisms that are capable of repairing H_2_O_2_-induced DNA damage in a timely manner. As assessed by % tail DNA and % tail length, DNA health seems to return to normal or near-normal levels 3.5 hours after the cessation of H_2_O_2_ treatment. Interestingly, preincubation with PBN or AA resulted in a significant increase in % tail length in ADSCs from donors aged 41-60 years or donors aged 61+ years (Fig. 5). Specifically, at the 4 hour timepoint, ADSCs pretreated with AA or PBN displayed a significantly higher % tail length than ADSCs treated with H_2_O_2_ alone. This finding was unexpected and stood out in contrast to our other data for PBN and AA. Although anomalous, it raises the possibility that AA and PBN may increase DNA damage under specific circumstances. This is not wholly unfeasible, as AA has been reported to exert both anti-oxidant and pro-oxidant effects [37, 38].

We initially hypothesized that ADSCs derived from older donors would show higher rates of DNA damage in response to H_2_O_2_ or UV light. Aging is a highly complex, multifaceted process characterized by the progressive decline in physiological integrity. Both genomic instability and stem cell exhaustion are two established hallmarks of aging [20, 39, 40]. Given this, we were surprised to find that age of the MSC donor failed to significantly impact tail length, tail DNA, or OTM in ADSCs subjected to genotoxic stress. DNA damage and repair in ADSCs from donors aged 61+ years were statistically comparable to ADSCs from donors aged 20-40 years and ADSCs from donors aged 41-60 years. While aged stem cells have been reported to exhibit decreased therapeutic potential and age-related decline [41-44], our data would suggest that DNA repair mechanisms in ADSCs remain robust with age. Aging-related complications in MSCs are therefore likely due to defects in other parameters besides H_2_O_2_-associated DNA repair.

Although our data demonstrate that treatment with either PBN or AA decreases genotoxic stress in cultured ADSCs, it would be of interest to learn of other agents that could also reduce levels of DNA damage. For examine, rapamycin has been reported to reverse senescence and improve immunoregulation in murine MSCs [45], making it a good candidate for a genotoxic protector. Adult stem cell-derived growth differentiation factor 6 has been reported to reduce age-related tissue dysfunction [46] and may similarly work to reduce DNA damage levels in ADSCs. Additional studies and screens are warranted to determine how ADSCs can be optimally protected against genotoxic stress prior to transplantation.

In conclusion, we illumine the basal rates of DNA damage and repair in human ADSCs subjected to genotoxic stress. We also identify two agents capable of reducing H_2_O_2_-induced DNA damage in ADSCs derived from differently aged donors. Our data raise the possibility that preincubation with either PBN or AA may help to maximize the genomic integrity and therapeutic efficacy of MSCs. Future studies should aim to understand the specific mechanisms by which PBN and AA reduce H_2_O_2_-induced DNA damage in ADSCs.

## MATERIALS AND METHODS

### Isolation of mesenchymal stem cells from adipose tissue

Human subcutaneous adipose tissues from different age groups and donors were obtained from Berufsgenossen-schaftliche Kliniken Bergmannstrost in Halle, Germany. Approval to perform the study was obtained by the Ethical Commission of the University of Halle and written consent forms were obtained by the donors.

In a manner analogous to our recently published work [47], adipose tissue was cut into 1-2 mm pieces under the laminar hood and then transferred into a 15 mL tube containing an equal volume of collagenase I (200 U/mL) / dispase (30 U/mL). Adipose tissue was digested in a 37°C water bath with intermittent shaking for 30-60 min. After the incubation time, an equal volume of culture media was added and centrifuged at 1000 rpm for 5 min. As a result of the digestion, three layers are visible after centrifugation. The first layer contains adipose tissue, the second layer consists of enzymes, and the third layer includes the stromal vascular fraction (SVF), which consists of stem cells and endothelial cells. The first and second layers were carefully removed with a pipette and 10 ml of culture media was added to the layer containing the SVF. The SVF was sieved using a 40 μm nylon mesh into a 50 mL tube. The filtered supernatant, which contains ADSCs, was centrifuged at 1000 rpm for 5 min. The supernatant was removed and 10 mL of growth media was added and transferred into a T75 flask.

### Culture of mesenchymal stem cells

Similarly to our previous work [47], ADSCs were cultivated in Dulbecco's modified Eagle's medium (DMEM, 1 g/L D-Glucose; Invitrogen) containing 10% fetal bovine serum (FBS; Hyclone) and 1% penicillin/streptomycin (Invitrogen). Culture media was changed every 3 days until cell proliferation was visible. ADSCs were trypsinized at 80% confluency. For the DNA damage experiments, ADSCs at passages 3-5 were used. ADSCs were categorized into 3 age groups: young (20-40 yrs), middle (41-60 yrs) and aged (61+ yrs).

### Induction of DNA damage with UV light

40,000 cells were seeded in a 12-well plate 24 hr prior to the beginning of the treatment. The next day, plates were placed under laminar flow and exposed to UV light for 30 min. As negative control (timepoint 0 hr), ADSCs were maintained in the incubator without exposure to UV light. Initial DNA damage was defined as damage at the 30 min timepoint while end DNA damage was defined as damage at the 4 hr timepoint.

### Induction of DNA strand brakes with H_2_O_2_

ADSCs were seeded 24 hr prior to treatment with H_2_O_2_ in a 12-well plate seeded at 40,000 cells per well. The culture media was removed and the cells were incubated with 0.4 mM of H_2_O_2_ in PBS for 30 min at 37°C. Afterwards, the H_2_O_2_ solution was replaced with culture media and the cells were placed at 37°C for 2 hr or 4 hr to allow for DNA repair. The negative control (timepoint 0 hr) was treated with PBS only. Initial DNA damage was defined as damage at the 30 min timepoint while end DNA damage was defined as damage at the 4 hr timepoint.

### Treatment with PBN and AA

Pre-conditioning of ADSCs with either PBN or AA was performed 24 hr prior to the induction of DNA damage. 40,000 ADSCs were seeded in a 12-well plate in culture media containing 800 μM PBN (Sigma-Aldrich) or 100 μM AA (Sigma-Aldrich). The next day, ADSCs were treated with H_2_O_2_ and, after incubation, the media was replaced with culture media containing PBN or AA to allow for repair.

### Alkaline Single Cell Gel Electrophoresis (Comet Assay)

DNA damage was assessed via the comet assay [48]. Microscopic slides were dipped into a vertical jar containing 1% agarose. The excess of agarose was removed and slides were dried at RT. Cells were embedded in low melting point (LMP) agarose. A cell concentration between 1-1.5 × 105 cells in 250 μL PBS was added to 750 μL of 1.3% LMP agarose in PBS at 37°C. A 100 μL volume containing the cells was then added to the 1% agarose slide and covered with a coverslip. Slides were incubated for 5 min at 4°C to allow for agarose solidification. The coverslip was removed and the cells lysed for 1 hr at 4°C with lysis buffer, pH 10 (2.5 M NaCl, 0.1 M EDTA-Na2, 10 mM Tris, and 1% Triton-X). After cell lysis, the DNA was unwound by alkaline treatment in cold electrophoresis solution, pH 13 (0.03 M NaOH and 2 mM EDTA-Na2) for 40 min at 4°C. The slides were then placed in an electrophoresis chamber and immersed in cold electrophoresis solution. Then, the DNA was run at 25 V for 30 min. Slides were then washed 3X with distilled water for 5 min at RT and the DNA was stained with 2.5 μg/mL of propidium iodide. Pictures were taken using a Leica system at a 20X magnification and the comets were analyzed with Comet Score software (TriTek CometScore Freeware v1.5). 50-100 comets were analyzed per slides.

### Statistics

4-6 ADSC donors (n = 4-6) were used for the experiments in this study. The data are presented as mean ± standard error of the mean (SEM). One-way ANOVA in conjunction with Tukey's test was used to determine statistically significant differences. Significance was defined as p < 0.05.
